# Editorial: Genetics and biomarkers of Alzheimer's disease in Asian populations

**DOI:** 10.3389/fnins.2024.1357783

**Published:** 2024-01-23

**Authors:** Xiaopu Zhou, Kin Y. Mok, Amy K. Y. Fu

**Affiliations:** ^1^The Centre for Applied Genomics, The Hospital for Sick Children, Toronto, ON, Canada; ^2^Genetics and Genome Biology Program, The Hospital for Sick Children, Toronto, ON, Canada; ^3^Division of Life Science, State Key Laboratory of Molecular Neuroscience and Molecular Neuroscience Center, The Hong Kong University of Science and Technology, Kowloon, Hong Kong SAR, China; ^4^Hong Kong Center for Neurodegenerative Diseases, Hong Kong Science Park, Hong Kong, China; ^5^Department of Neurodegenerative Disease, UCL Institute of Neurology, London, United Kingdom; ^6^Guangdong Provincial Key Laboratory of Brain Science, Disease and Drug Development, HKUST Shenzhen Research Institute, Shenzhen, Guangdong, China

**Keywords:** Alzheimer, biomarker, Asian, ATN, mass-spec, Mendelian randomization, diagnosis, stratification

Alzheimer's disease (AD) is a significant global health concern, with its impact amplified by the aging population. Researchers have been working to unravel the intricate mechanisms underlying this inheritable and age-related neurodegenerative disorder, with great efforts have been directed toward devising tools for early detection and risk assessment during its initial stages. Recent strides in genetic research have illuminated key genetic variants implicated in AD pathogenesis (Kunkle et al., 2019), while fluid biomarker investigations have revealed specific proteins reflecting disease severity in cerebrospinal fluid (CSF) and plasma (Leuzy et al., 2021).

However, it's important to note that much of this research has been concentrated within cohorts of European ancestry. Non-European descent populations, with their different genetic and lifestyle characteristics, have been underrepresented in these investigations (Zhou et al., 2021). Considering these differences, alternative genetic risk factors might play a role in the development of AD in these populations. A recent transethnic AD genome-wide association study identified ancestry-specific loci, underscoring the need for more research into non-European populations' genetics, biomarkers, and mechanisms (Rajabli et al., 2023).

Notably, Asian populations constitute a substantial proportion of the global population. To bridge the gap, we initiated “*Genetics and biomarkers of Alzheimer's disease in Asian populations*” Research Topic in January 2022. This initiative aimed to spotlight AD and dementia research specifically in Asian populations, fostering collaboration and sharing research findings. By June 2022, this effort yielded 12 manuscripts, with five making it into the Research Topic. These contributions spanned various ethnic origins, involving participants from Iran, China, and Japan. They also covered diverse research approaches, including biomarkers analysis in cerebrospinal fluid, plasma, and urine, as well as brain transcriptome analysis and Mendelian randomization.

In particular, study led by Asadi et al. delved deep into the role of RNA-binding proteins within AD, presenting potential biomarkers including BDNF and Tristetraprolin (TTP) that emerge from blood transcript level analysis in the Iranian population. In parallel, Sun et al. meticulously examined the correlation between plasma biomarkers and AD in a Chinese cohort. Their findings underscored the diagnostic potential of specific protein markers including TNF-a and plasma ATN biomarkers (abeta42/40 ratio, p-tau181, and NfL) (Sun et al.). Meanwhile, Chen et al. utilized liquid chromatography and mass spectrometry to meticulously distinguish urine biomarkers for vascular dementia (VD) from other conditions. Impressively, they pinpointed a set of 18 proteins with the capability to effectively classify VD from both AD and normal control groups in the Chinese population (Chen et al.). Gan et al.'s study further enriched our understanding by investigating the intricate interplay between blood-brain barrier (BBB) permeability and dementia in a Chinese cohort. They illuminated potential connections involving the CSF/serum albumin ratio (Qalb) – a marker of BBB permeability – and vascular risk factors also AD ATN CSF biomarkers (Gan et al.). Lastly, Zhu et al. explored the intricate relationship between smoking and AD within Chinese and Japanese cohorts. Employing Mendelian randomization analysis, they rigorously assessed whether any causal connection existed between smoking and AD risk. Interestingly, their comprehensive findings unveiled no significant association between smoking and AD (Zhu et al.).

Significantly, a substantial portion of these studies have delved deeply into exploring and harnessing the potential of biomarkers. With the swift advancements in biotechnology, the prospect of clinically accessible biomarker solutions for diagnosing AD is becoming increasingly plausible (Blennow and Zetterberg, 2018). Specifically, the highly sensitive proteome platform facilitates precise detection of neurodegeneration markers in the blood (Hampel et al., 2018; Hansson et al., 2022). Complementarily, high-throughput proteome methods enhance the accuracy of disease diagnosis and staging by establishing a scoring system based on multi-biomarker panels (Jiang et al., 2022). Besides proteome analysis, emerging biomarkers from various sources, such as imaging analysis (Liu et al., 2018), transcriptome (Zhong et al., 2021), metabolome (Green et al., 2023), and gut microbiota (Jiang et al., 2017), hold promise for aiding AD diagnosis. These diverse methods address distinct aspects of pathophysiological changes in patients throughout the course of the disease, potentially reshaping future clinical practices in AD diagnosis.

Nonetheless, extending beyond accurate diagnosis, the potential to revolutionize Alzheimer's disease (AD) lies in tools and methods for early risk prediction and patient stratification. Consider, for instance, the utilization of imaging (Li et al., 2019), polygenic risk analysis (Zhou et al., 2023), and fluid biomarkers (Palmqvist et al., 2021) to forecast AD risk. These applications must undergo rigorous testing and optimization within general populations before being integrated into routine clinical practices. Furthermore, the correlation between the *APOE*-ε4 genotype and ARIA symptoms in AD patients undergoing monoclonal antibody therapy underscores the importance of improved patient stratification during drug development and disease intervention (VandeVrede et al., 2020). While undeniably pivotal, the development of such strategies necessitates meticulous analyses conducted within well-designed AD cohorts, backed by multi-omics data and meticulously documented clinical information. Such comprehensive resources would empower us to map the onset and progression of AD on a population scale, enabling a deeper comprehension of its underlying causes and facilitating clinical research aimed at early intervention solutions.

Recognizing the diverse medical practices across global populations, we foresee the necessity for concerted endeavors aimed at establishing regional AD cohorts. The precedent set by Alzheimer's Disease Neuroimaging Initiative (ADNI) stands as a testament to the success that can be achieved through such an approach, fostering fruitful research outcomes (Petersen et al., 2010). This proactive approach would enable us to address AD diagnosis and interventions in a systemic manner, while also considering the unique contexts of each society. It holds the potential to elevate AD diagnosis and interventions to a more comprehensive systemic perspective, promising the emergence of more encompassing and impactful outcomes in the near future.

## Author contributions

XZ: Writing—original draft, Writing—review & editing. KM: Writing—original draft, Writing—review & editing. AF: Writing—original draft, Writing—review & editing.
